# Posterior reversible encephalopathy syndrome with Lilliputian hallucinations secondary to Takayasu's arteritis

**DOI:** 10.1016/j.radcr.2020.07.080

**Published:** 2020-08-24

**Authors:** R.M. Dunne, J. Duignan, N. Tubridy, L. O'Neill, J.A. Kinsella, T.A. Omer, G. McNeill, R.P. Killeen

**Affiliations:** a Department of Radiology, St. Vincent's University Hospital, Elm Park, Dublin, Ireland; b Department of Neurology, St. Vincent's University Hospital, Elm Park, Dublin, Ireland; c Department of Rheumatology and General Medicine, St. Vincent's University Hospital, 196 Merrion Rd, Elm Park, Dublin, D04T6F4, Ireland

**Keywords:** Posterior reversible encephalopathy syndrome, Takayasu's arteritis

## Abstract

Posterior Reversible Encephalopathy Syndrome (PRES) is a rare complication of Takayasu's Arteritis. A 54-year-old, right-handed woman presented with Lilliputian visual hallucinations, postprandial abdominal pain, blurred vision and headaches. She then had a tonic-clonic seizure. Neuroimaging revealed characteristic white matter oedema of the occipital lobes, in keeping with PRES. Renal infarcts and abnormalities of the abdominal aorta, subclavian, mesenteric, and internal carotid arteries were demonstrated on further imaging. The combination of hypertension, absent peripheral pulses, postprandial claudication, and imaging abnormalities of the aorta as well as its branches, lead to the diagnosis of PRES secondary to Takayasu's Arteritis. Treatment with oral steroids resulted in complete resolution of the patient's symptoms and abnormalities found on CT and MRI brain imaging. Takayasu's Arteritis is a rare vasculitis, more common in women and PRES is an unusual complication. Symptoms of PRES may include headache, seizures, hallucinations, confusion, and altered consciousness. Risk factors for PRES include; pregnancy, immunosuppression, renal disease, hypertension and rheumatological disorders. Vasogenic oedema in affected lobes, most often occipital, is characteristic of PRES on neuroimaging. Prompt treatment of PRES can avoid catastrophic consequences such as death and can achieve complete resolution of symptoms and imaging abnormalities.

## Introduction

**Fig. 1:**
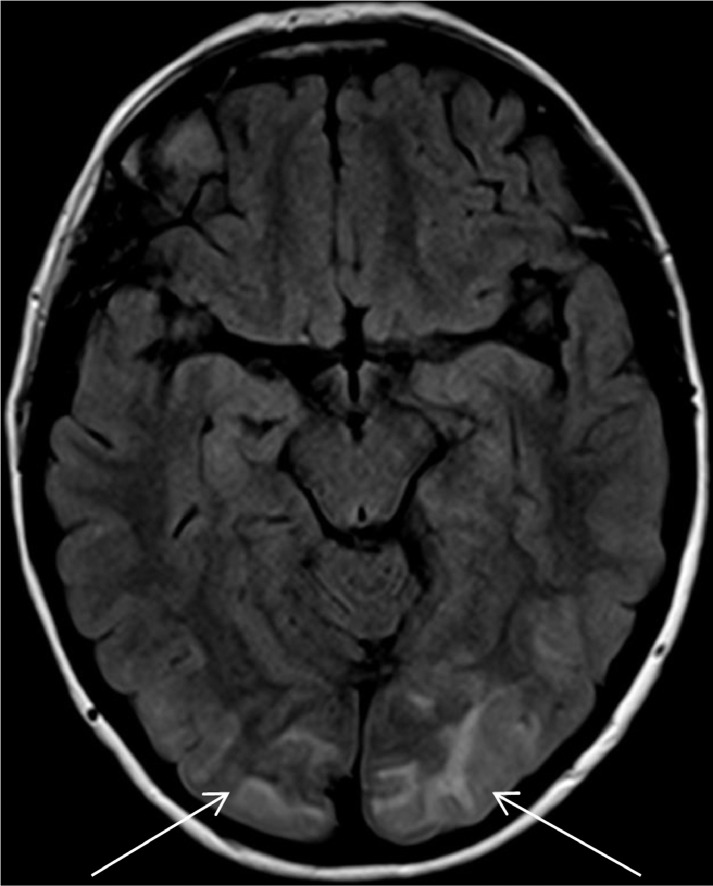
Axial FLAIR MRI brain demonstrating high signal within the subcortical white matter of both occipital lobes.

**Fig. 2:**
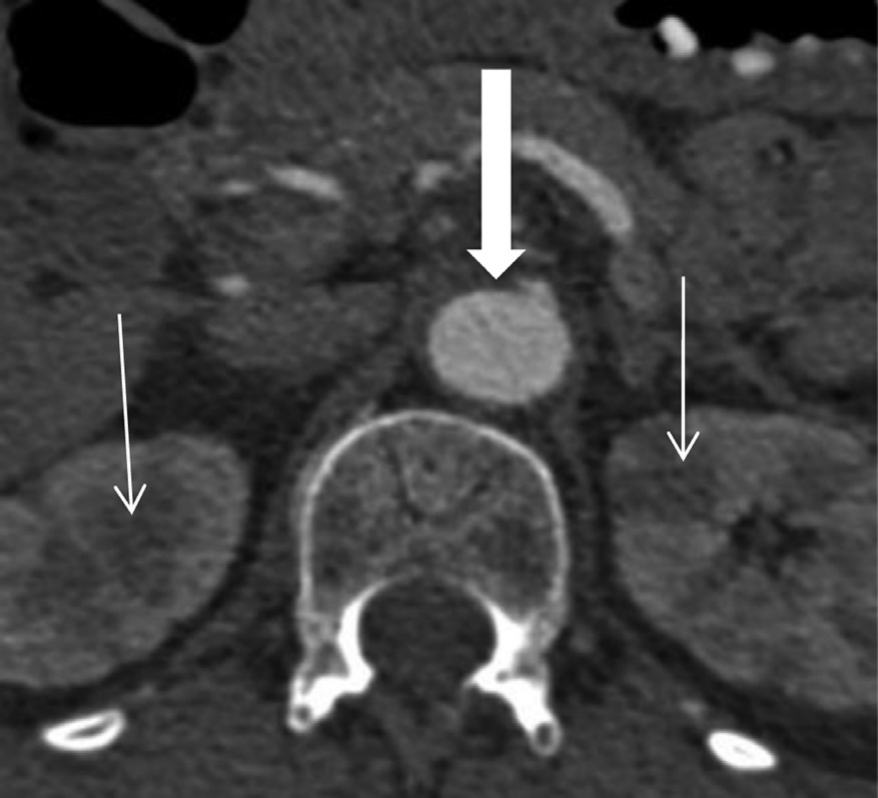
Axial CT Abdomen demonstrating renal infarcts and mural thickening of the aorta at the level of the celiac axis (block arrow).

**Fig. 3:**
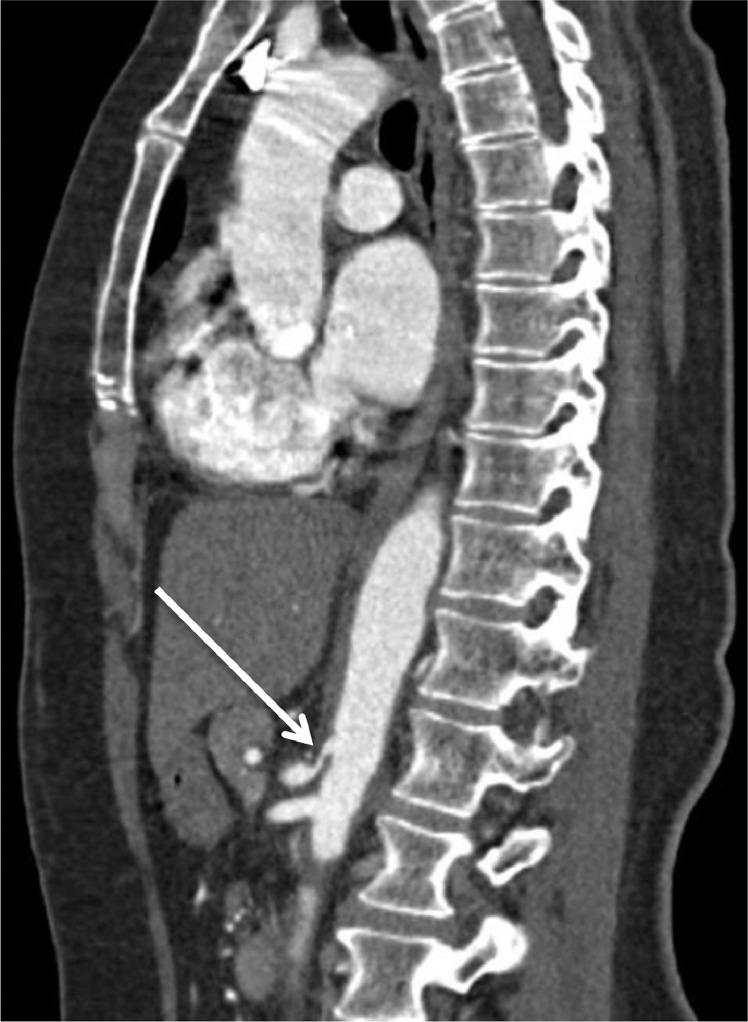
Sagittal CT of the aorta showing thickening of the wall of the aorta at the level of the celiac axis and superior mesenteric artery.

**Fig. 4:**
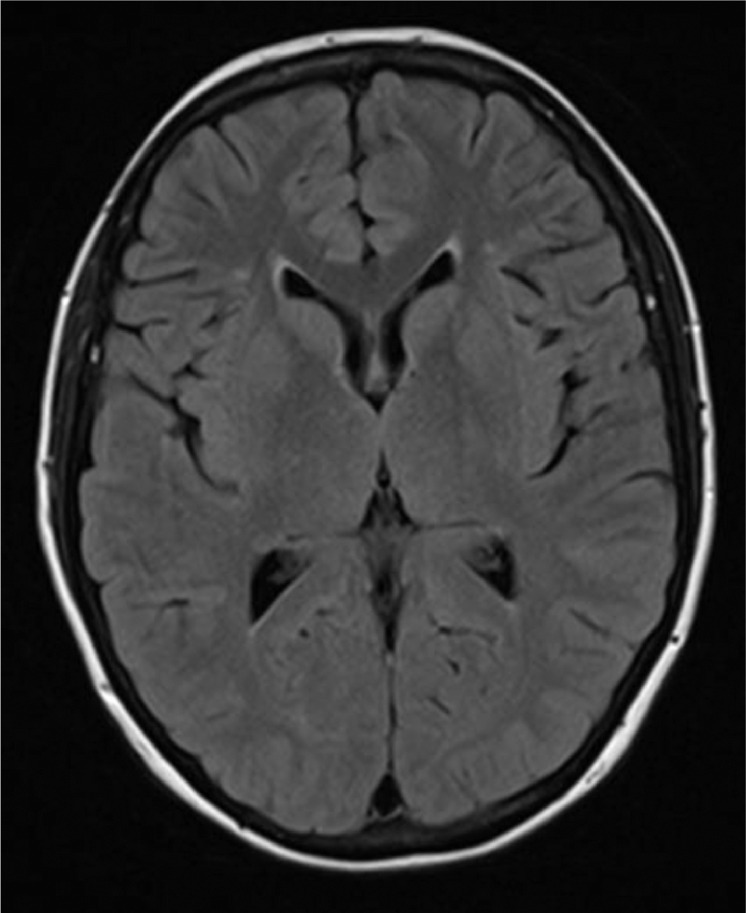
Follow-up axial FLAIR MRI brain demonstrating interval resolution of the white matter signal abnormality.

This case history outlines the presentation of a 54-year-old female with signs and symptoms of Posterior Reversible Encephalopathy Syndrome (PRES), secondary to Takaysu's Arteritis. PRES is a rare complication of Takayasu's Arteritis [2]. PRES, generally presents with symptoms of headaches, seizures, visual disturbances, confusion, or altered consciousness [2,^4^,7]. Vasogenic oedema in the occipital, parietal, frontal and temporal lobes, is characteristic of PRES on neuroimaging [7]. Prompt diagnosis and appropriate treatment of PRES can achieve full resolution of symptoms and abnormalities found on neuroimaging [6,7]. Early diagnosis and treatment of PRES is important to prevent and avoid complications such as hemorrhage, hydrocephalus, brainstem compression, and death [8]. Risk factors for PRES include; pregnancy, immunosuppression, hypertension and rheumatological disorders [4,7]. Clinicians should consider the diagnosis of PRES, in patients with risk factors, presenting with symptoms such as headache, seizure, altered consciousness, or visual disturbance (Fig. 1, Fig. 2, Fig. 3, Fig. 4).

## Case Presentation

A 54-year-old female presented to an Emergency Department with abdominal pain. She described a 3-week history of worsening postprandial abdominal pain, intermittent generalized headache, blurred vision, and visual hallucinations including Lilliputian imagery. Lilliputian hallucinations are visual hallucinations of people, objects, or animals that appear reduced in size and can occur secondary to a variety of metabolic and neurological conditions [11]. This patient described small men and women dressed in Dickensian costumes. Her past medical history was significant for Raynaud's Syndrome.

As part of her work-up, a CT brain scan was arranged and during the scan, she had a generalized tonic-clonic seizure, which was treated with 4 mg of intravenous (IV) lorazepam, followed by 1 g of IV levetiracetam. Subsequently, her Glasgow Coma Score was recorded as 9/15 but neurological examination showed no clear localizing signs at this point. She was noted to be hypertensive 160/100 mmHg. Peripheral pulses were noted to be absent on examination. The patient remained postictal with associated confusion, which subsequently resolved after a few hours.

Differential diagnoses at this point included:

• Viral encephalitis
• Ischemic stroke
• Toxin exposure ie, substance misuse/withdrawal or poisoning
• Space occupying lesion
• Metabolic encephalopathy eg, hypoglycemia
• Systemic or intra-abdominal infection
• Encephalopathy secondary to other cause.

A series of investigations were then performed to establish a diagnosis and cause for the patient's symptoms. Blood results revealed a leucocytosis 14.3 × 10^2^/l (normal range 4-11), a neutrophilia 11.6 × 10^2^/L (2-8) and an elevated CRP 125 (<10). A vasculitic screen produced a positive smooth-muscle antibody and a weakly positive ANCA. Lumbar puncture revealed a normalCSF white cell count, CSF glucose 3.5 mmol/L (2.5-4, no paired serum glucose) and a slightly elevated CSF protein 0.48 g/L (0.15-0.45) with a negative viral PCR including HSV-1. The elevated CRP and weakly positive ANCA results were consistent with an inflammatory process. Serum creatinine and estimated glomerular filtration rate was within normal limits but urinary protein:creatinine ratio was elevated 23 mg/mmol (3-14) indicating microalbuminuria.

A CT brain scan demonstrated areas of low attenuation in the occipital lobes, in keeping with possible PRES. CT angiogram of intracranial, extracranial vessels, and aortic arch demonstrated irregularity of the left subclavian and internal carotid arteries as well as stenosis of the left vertebral artery. A CT thorax, abdomen and pelvis revealed multiple, bilateral, renal infarcts, and thickening of the abdominal aorta and its branches, indicating a large vessel vasculitis. An MRI brain study showed bilateral subcortical hyperintensities in the occipital lobes, again consistent with PRES. Ultrasound of her temporal arteries did not reveal any abnormality, with no evidence of Giant Cell Arteritis.

The patient was commenced on reducing oral prednisolone, starting at 30 mg once daily. This resulted in marked improvement in confusion, headache, and resolution of the visual hallucinations. Following this, an increasing dose of mycophenolate moftil was prescribed, in addition to levetiracetam 500 mg twice daily and amlodipine 5 mg once daily. Prophylaxis against Pneumocystis Jiroveci Pneumonia using trimethoprim/sulfamethoxazole was also provided.

Throughout the remainder of her hospital stay, the patient remained well without further seizures and was alert and orientated. Her visual hallucinations, confusion, headache and blurred vision completely resolved. A follow-up MRI brain study performed 12 days later, showed resolution of PRES findings. The patient was discharged well and asymptomatic.

## Discussion

Takayasu's Arteritis is an uncommon vasculitis, primarily affecting the aorta and its branches [2]. It is most prevalent amongst females aged 10-30 years, [1,2] and Takayasu's Arteritis is more common in people of Asian ethnicity [9]. PRES is a rare complication [3]. A diagnosis of Takayasu's Arteritis can be made when 3 of the following 6 criteria are present; onset under 40 years, claudication of an extremity, reduced peripheral pulse, discrepancy of blood pressure recordings between upper limbs, bruit over the aorta or subclavian arteries and/or evidence of narrowing of the aorta or its primary branches [10]. We found 12 other cases of PRES secondary to Takayasu's Arteritis in the literature.

PRES typically presents with headaches, seizures, visual disturbances, confusion, altered consciousness, and characteristic findings on neuroimaging [2,^4^,7]. PRES is often associated with hypertension, renal disease, rheumatological conditions, and some immunosuppressive medications [4,^6^,7]. Typical findings on MRI brain imaging show vasogenic oedema in the affected lobes, [5,7] most frequently the occipital lobes, followed by the parietal, frontal, and temporal lobes [7].

Full resolution of symptoms and neuroimaging abnormalities, can be achieved within 2-3 weeks, if the condition is diagnosed and treated promptly [6,7]. Without recognition and appropriate treatment, catastrophic complications, including death, have been reported [8]. Death can occur as a result of hemorrhage, hydrocephalus, or brainstem compression [8]. Treatment is focused on control of hypertension and seizures and on treating the underlying cause, eg, withdrawal of immunosuppression or as outlined here, treatment of the underlying vasculitis.
